# Differential Regulation of the *Period* Genes in Striatal Regions following Cocaine Exposure

**DOI:** 10.1371/journal.pone.0066438

**Published:** 2013-06-11

**Authors:** Edgardo Falcon, Angela Ozburn, Shibani Mukherjee, Kole Roybal, Colleen A. McClung

**Affiliations:** 1 Department of Psychiatry, University of Texas Southwestern Medical Center, Dallas, Texas, United States of America; 2 Department of Psychiatry and Translational Neuroscience Program, University of Pittsburgh School of Medicine, Pittsburgh, Pennsylvania, United States of America; University of Chicago, United States of America

## Abstract

Several studies have suggested that disruptions in circadian rhythms contribute to the pathophysiology of multiple psychiatric diseases, including drug addiction. In fact, a number of the genes involved in the regulation of circadian rhythms are also involved in modulating the reward value for drugs of abuse, like cocaine. Thus, we wanted to determine the effects of chronic cocaine on the expression of several circadian genes in the Nucleus Accumbens (NAc) and Caudate Putamen (CP), regions of the brain known to be involved in the behavioral responses to drugs of abuse. Moreover, we wanted to explore the mechanism by which these genes are regulated following cocaine exposure. Here we find that after repeated cocaine exposure, expression of the *Period (Per)* genes and *Neuronal PAS Domain Protein 2 (Npas2)* are elevated, in a somewhat regionally selective fashion. Moreover, NPAS2 (but not CLOCK (Circadian Locomotor Output Cycles Kaput)) protein binding at *Per* gene promoters was enhanced following cocaine treatment. Mice lacking a functional *Npas2* gene failed to exhibit any induction of *Per* gene expression after cocaine, suggesting that NPAS2 is necessary for this cocaine-induced regulation. Examination of *Per* gene and *Npas2* expression over twenty-four hours identified changes in diurnal rhythmicity of these genes following chronic cocaine, which were regionally specific. Taken together, these studies point to selective disruptions in *Per* gene rhythmicity in striatial regions following chronic cocaine treatment, which are mediated primarily by NPAS2.

## Introduction

Drug addiction is associated with major disruptions in circadian rhythms. For example, drug addicts are commonly reported to have disruptions in their sleep/wake cycle, activity cycles, eating habits, as well as, blood pressure, hormone secretion and body temperature rhythms [1], [2]. Even though the master pacemaker is located in the Suprachiasmatic Nucleus (SCN), circadian genes and proteins that make up the molecular clock are widely expressed throughout the brain, thereby forming SCN-independent pacemakers that entrain to other non-photic stimuli such as food [3], [4]. Drugs of abuse can also serve as powerful Zeitgebers for some of these clocks outside of the SCN. Genes that are important in regulating drug-induced behaviors are often induced or repressed throughout striatal regions by drugs of abuse [5]. Indeed, various studies have found changes in the expression of circadian genes in striatal regions in response to psychostimulants. Interestingly, these changes are often specific to a given region or treatment. For example, *rPer1* is induced in the rat caudate-putamen (CP) following acute cocaine, while *rPer2* is induced following a chronic “binge” pattern of cocaine [6]. Another study showed that acute methamphetamine treatment leads to a rapid induction of *mPer1*, but not *mPer2* or *mPer3* expression in the mouse CP [7]. This suggests that the induction of circadian genes in these regions is specific, or that *mPer1* is rapidly induced by cocaine while the induction of the other *Per* genes lags behind. Indeed, rapid induction of *mPer1* is seen in the SCN in response to light suggesting a distinct molecular mechanism that underlies its regulation [8]. Furthermore, a study by Lynch *et al* found that seven days of cocaine self-administration lead to a significant upregulation of a number of circadian and circadian-associated genes in the dorsal striatum, including *Clock, Brain and Muscle ARNT-like protein 1 (Bmal1), Per2,* and *Cryptochrome1 (Cry1)*, among others [9]. These studies suggest that psychostimulants have selective effects on the expression of these genes in striatal regions. However, it is important to note that many, if not all, of these previous studies have looked at expression of circadian genes at only a specific timepoint, when in fact the *Per* genes are known to have rhythmic and different temporal profiles of expression in limbic regions [10]. Thus, it is also important to examine the expression of these genes throughout a 24 hour period in order to distinguish changes in rhythm amplitude or phase from specific drug-induced timepoint-dependent effects.

Studies in several model organisms have implicated the genes that make up the circadian clock in the behavioral responses to drugs of abuse. Pioneering studies performed in *Drosophila melanogaster* showed that flies lacking a functional *Per, Clock, Cycle* or *Doubletime* gene all fail to sensitize to cocaine [11]. Later, Abarca *et al* found that *mPer1^Brdm1^* knock-out mice fail to sensitize to cocaine and show no conditioned preference for cocaine, while *mPer2^Brdm1^* knock-out mice show a hyper-sensitization to cocaine, but normal levels of conditioned preference for cocaine, suggesting a regulatory role for the *Per* genes in cocaine-associated behaviors [12]. However, a recent study by the same group found that the *mPer1^Brdm1^* knock-out mice were indistinguishable from wild-type littermates in terms of their inclination to self-administer cocaine and reinstate their cocaine-seeking behavior [13]. Furthermore, the *mPer1^Brdm1^* mice display normal levels of alcohol self-administration and reinstatement of alcohol-seeking behavior, while the *mPer2^Brdm1^* mice show an increase in alcohol self-administration [14], [15]. Studies have also shown that morphine reward is regulated by the *Per* genes. *mPer2^Brdm1^* mutant mice fail to develop tolerance to the analgesic effects of morphine when tested on hot plate or tail-immersion tests [16]. Moreover, these mice also showed a suppression of morphine-induced withdrawal syndrome when compared to their wild-type counterparts [16]. *mPer1* has also been implicated in the regulation of the rewarding effects of morphine [17]. A reduction in *mPer1* via a DNAzyme blocks conditioned preference for morphine, and this effect may involve the ERK signaling pathway [17], [18]. Thus, both *mPer1* and *mPer2* are involved in regulating the behavioral and physiological responses to drugs of abuse; however their roles in mediating these processes seem to depend on which drug is used and the behavior being studied.

Both NPAS2 and CLOCK can heterodimerize with BMAL1 to activate transcription of the *Per* and *Cry* genes among other clock-controlled genes [19]. However, while CLOCK is widely expressed throughout the brain, including high levels in the SCN, the expression of NPAS2 is restricted to the forebrain, with very low expression in the SCN under normal conditions [20]–[22]. NPAS2 has little to no expression in the ventral tegmental area (VTA), but it has high levels of expression in both CP and nucleus accumbens (NAc), which receive input from midbrain dopaminergic regions. Therefore NPAS2 may be involved in regulating the response to drugs of abuse, as well as, the regulation of *Per* gene expression in the striatum. This study investigated the differential regulation of the *Per* genes by cocaine, the role of NPAS2 and CLOCK in this regulation, and the effect of repeated cocaine injections on molecular rhythms of circadian genes in striatal regions.

## Materials and Methods

### Animals

C57BL/6J mice (The Jackson Laboratory) and *Npas2* mutant mice [22] were group housed in a 12/12 light/dark (LD) cycle (lights on at 7am, lights off at 7pm) with food and water *ad libitum*. For the 24-hr time series studies, mice were group housed under the same LD schedule in temperature-controlled and sound-proof cabinets. Homozygous *Npas2* mutant mice and wild type littermates were produced by heterozygous breeding. Male, 8-week old mice were used in all studies. Ethics statement: All animal use was approved by the University of Texas Southwestern Medical Center Institutional Animal Care and Use Committee (IACUC protocol # 1098-07-01).

### Drug

Cocaine hydrochloride was generously provided by the National Institute on Drug Abuse. Cocaine was diluted in 0.9% NaCl and animals were injected with a 15 mg/kg cocaine or saline intraperitoneally (i.p.) at a volume of 2 ml/kg. Cocaine treatment was performed during ZT 4–6 and animals were sacrificed by decapitation 24 h after the last injection, as previously published [23].

### Quantitative real time PCR

RNA was isolated from mechanically homogenized tissue using the Trizol reagent (Invitrogen) according to the manufacturer’s instructions. This was followed by a 15 min treatment with DNAse I (Invitrogen) according to manufacturer’s protocols to digest any remaining genomic DNA. One microgram of total RNA was used to synthesize cDNA using Superscript III Reverse Transcriptase (Invitrogen) per the manufacturer’s instructions. cDNA or chromatin samples were mixed with SYBR Green master mix (Applied Biosystems, ABI, Grand Island, NY) and specific primers for genes or promoter regions of interest. Prior to the experiment primer sets were tested thoroughly to determine reaction efficiency, specificity, and the absence of primer-dimers. Primers used were: *Clock* Forward 5′-CAGAACAGTACCCAGAGTGCT-3′, Reverse 5′-CACCACCTGACCCATAAGCAT-3′; *Npas2* Forward 5′-GACACTGGAGTCCAGACGCAA-3′, Reverse 5′-AATGTATACAGGGTGCGCCAAA-3′; *mPer1* Forward 5′-CTCTGTGCTGAAGCAAGACCG-3′, Reverse 5′-TCATCAGAGTGGCCA GGATCTT-3′; *mPer2* Forward 5′-GAGTGTGTGCAGCGGCTTAG-3′, Reverse 5′-GTAGGGTG TCATGCGGAAGG-3′; *mPer3* Forward 5′-GTCCATCTGGAGAATGATAGAGCG-3′, Reverse 5′-GCTTCAGCACCTCCTCTCGAC-3′; *Gapdh* Forward 5′-AACGACCCCTTCATTGAC-3′, Reverse 5′-TCCACGACATACTCAGCAC-3′; *Cyclophilin* Forward 5′-CATCTATGGTGAGCGCTTCCCA-3′, Reverse 5′-GCCTGTGGAATGTGAGGGGTG-3′. Reactions were run on an ABI Prism 7700 real-time PCR machine. Fold changes and relative gene expression were calculated using the comparative Ct method and normalized to the corresponding *Gapdh* and *Cyclophilin* mRNA levels. The Ct values used for these calculations are the mean of at least four biological replicates of the same reaction; each PCR reaction was also performed in duplicate.

### Western Blots

Western Blot assays were run as previously published [24]. After chronic cocaine treatment, tissue punches were taken and frozen at -80°C. Brain tissue was sonicated on ice in a modified detergent based buffer containing both phosphatase and protease inhibitors (Roche, San Francisco, CA; Sigma, St. Louis, MO). After sonication, samples were denatured in boiling water and centrifuged at 15,000xg for 15 min, supernatant was subsequently collected and processed; protein concentration amounts were then quantified using a Bradford assay (Bio-Rad, Hercules, CA). Samples were run on a 10% acrylamide/bisacrylamide gel, transferred to a PVDF membrane, blocked in 5% milk and incubated with primary antibodies (PER 1,2,3 Chemicon, Temecula, CA). Blots were subsequently visualized using a chemiluminescence system (Pierce, Rockford, IL). All samples were normalized to GAPDH (Fitzgerald, Concord, MA). Standard curves were performed to ensure that the assay was conducted in the linear range. Densitometry was conducted using NIH image J software.

### Chromatin Immunoprecipitation (ChIP)

Brain tissue was processed as previously reported [25]. NAc and CP brain punches were taken from 8 mice and pooled, and immediately cross-linked in 1% formaldehyde for 15 min at room temperature. The cross-linking reaction was stopped by adding glycine to a final concentration of 0.125 M. The tissue was washed 5 times in cold 1x PBS containing Complete Protease Inhibitor Cocktail (Roche, San Francisco, CA) and then frozen on dry ice. The chromatin was solubilized and extracted by detergent lysis, followed by sonication. First, fixed tissue was homogenized twice, for 10 sec, in a cell lysis buffer (10 mM Tris, 10 mM NaCl, 0.2% Nonidet P-40). Next, the extracted chromatin was sheared to roughly 500-1,000 bp using a Sonic Dismembrator 550 (Fisher, Hampton, NH). Each sample was sonicated 8 times on ice, 20 sec each, at 25% of maximum power. After the chromatin lysate was extracted and properly fragmented, the optical density of each sample was determined. Equal amounts of chromatin lysate, 60 µg, were diluted with ChIP dilution buffer (Upstate, Billerica, MA) to a final volume of 1.1 ml. 100 µl of the pre-immunoprecipitated lysate was saved as “input” for later normalization. The chromatin solution was pre-cleared with either salmon sperm DNA/protein A-agarose gel slurry (Thermo Scientific, Waltham, MA) or Protein G Agarose/Salmon Sperm DNA (Thermo Scientific, Waltham, MA) for 45 min at 4°C. It was then immunoprecipitated overnight at 4°C with an antibody directed against a specific protein either CLOCK H-276 X or NPAS-2 H-20 X (Santa Cruz Biotechnology, Santa Cruz, CA). As a negative control, samples were immunoprecipitated with non-immune rabbit IgG or Anti-acetyl-Histone H3 (Upstate, Billerica, MA). Following immunoprecipitation, the DNA-protein complex was collected with either 40 µl salmon sperm DNA/protein A-agarose beads or 50 µl Protein G Agarose/Salmon Sperm DNA for 2 hr. The beads were sequentially washed once with low salt, high salt, LiCl, and twice with TE buffers. The DNA-protein complex was then eluted from the beads with 500 µl NaHCO_3_/SDS elution buffer. Proteins were reverse-cross linked from DNA using Proteinase K (Invitrogen, Grand Island, NY) under high-salt conditions at 65°C for at least 4 hr. The DNA, associated with a particular transcription factor, was extracted with phenol/chlorophorm/isoamyl alcohol, precipitated with 100% ethanol, washed with 70% ethanol, and finally resuspended in 0.1X TE Buffer diluted in PCR-grade water. Levels of specific transcription factor binding or histone modifications at each gene promoter of interest were determined by measuring the amount of associated DNA by real-time PCR as described above. Input or total DNA (nonimmunoprecipitated) and immunoprecipitated DNA were amplified in duplicate in the presence of SYBR Green (ABI, Foster City, CA). Primers used were: *mPer1* Promoter Forward 5′-CCTCCTCTAAGGGAAACACCA-3′, Reverse 5′-GCAAGTGAAGAGGCCAACAC-3′, *mPer2* Promoter Forward 5′-GCAGCATCTTCATT GAGGAACC-3′, Reverse 5′-CTCCGCTGTCACATAGTGGAAAACGTGA-3′; *mPer3* Promoter Forward 5′-CATCTTAGGCTTTCTTGACTTTGAG-3′, Reverse 5′-CAGAGAGCAAGTATCCA CATTTCAT-3′. Relative quantification of template DNA was performed using the comparative Ct method.

### Data Analysis

Single timepoint qPCR studies, imunoblots, and ChIP assays were analyzed by Student’s t-test. For the time series experiments, one-way ANOVAs followed by Tukey’s Multiple Comparison Test were initially used to establish diurnal rhythmicity for each gene and each condition, as previously published [26]. Additionally, two-way ANOVAs followed by post-hoc t-tests were used to assess the overall effect of treatment and time. In all experiments p<0.05 is considered significant. All data are expressed as mean ± standard error of the mean.

## Results

### *Npas2* expression is induced by chronic cocaine in striatal regions while *Clock* and *Bmal1* expression is unchanged

**Figure 1:**
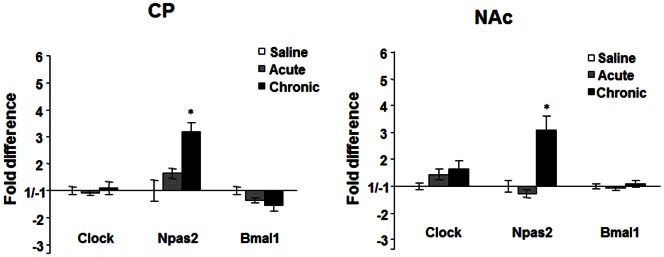
*Clock*, *Npas2* and *Bmal1* expression after cocaine treatment. Real-time PCR analysis of *Clock, Npas2,* and *Bmal1* expression in the CP and NAc following saline, acute (15 mg/kg, 1 day), or chronic cocaine treatment (15 mg/kg, 7 days) in wild type mice. *p<0.05 by t-test, n = 6.

To better understand the molecular actions of CLOCK and NPAS2 in striatal regions, we administered acute cocaine (15 mg/kg, i.p.), chronic cocaine (15 mg/kg, i.p., once/day for 7 days), or saline, and examined the expression levels of *Clock*, *Npas2*, and the binding partner for both genes, *Bmal1,* 24 hours after treatment (Zeitgeber time (ZT) 4). This chronic treatment paradigm has been used in previous studies to elicit cocaine sensitization and induces long term changes in cocaine responsive genes and proteins [27]. Interestingly, we found that *Npas2* mRNA expression was upregulated in both the NAc and CP following chronic but not acute

cocaine exposure (NAc: t = 2.4, p<0.05; CP: t = 2.5, p<0.05, Figure 1). Expression levels of *Clock* and *Bmal1* were not significantly altered by cocaine in these regions following these treatment paradigms (Figure 1). These results suggest that chronic cocaine treatment selectively affects the expression of *Npas2* in striatal regions.

### The *Period* genes are differentially induced by cocaine in striatal regions

**Figure 2:**
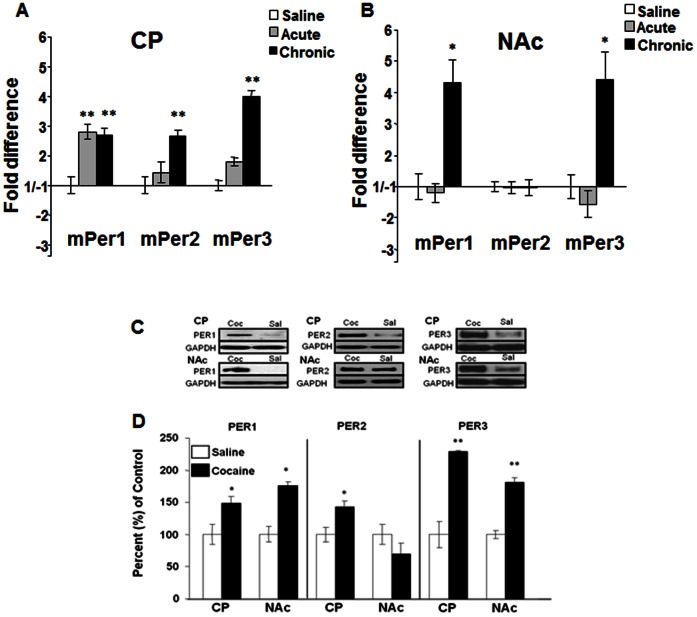
*Per* gene and protein expression after cocaine treatment. Real-time PCR analysis of *mPer1, mPer2* and *mPer3* expression in the CP (A) and NAc (B) following saline, acute (15 mg/kg, 1 day), or chronic cocaine treatment (15 mg/kg, 7 days) in wild type mice. *p<0.05, **p<0.01 by t-test. n = 6. (C, D) Cocaine (15 mg/kg) or saline was given chronically (7 days) i.p. Protein levels were measured 24 hrs later using western blot analysis in the CP and NAc. GAPDH was measured as a loading control. n = 5–8. Representative blots are shown (C) and the percent change in cocaine vs saline is shown (D). *p<0.05, **p<0.01 by t-test.

Both NPAS2 and CLOCK regulate the expression of the *Per* genes (*Per1and 2*) and *Cry* genes (*Cry1* and *2*) [19], [28]. These proteins serve as inhibitors of NPAS2/BMAL1 or CLOCK/BMAL1 function in transcriptional feedback loops throughout the brain [29]. There have been reports implicating *Per1* and *Per2* in the behavioral responses to cocaine, methamphetamine, morphine, and alcohol [7], [12], [15], [17], [30]–[32]. However, it appears that they may serve differential functions in response to these drugs. Therefore, we wanted to determine if these genes are differentially regulated by acute or chronic cocaine in the NAc or CP. We again administered acute cocaine (15 mg/kg, i.p.), chronic cocaine (15 mg/kg, i.p., once/day for 7 days), or saline and examined the expression levels of the *mPer1,mPer2 mPer3, Cry1,* and *Cry2* genes, 24 hours after treatment (ZT 4). Similar to previous studies that measured gene expression changes in the CP with psychostimulant exposure [6], [7], we found that only *mPer1* in the CP is regulated by acute cocaine (t = 3.05, p<0.01), however, all three *Per* genes were upregulated following chronic cocaine (*mPer1*: t = 3.00, p<0.01; *mPer2*: t = 3.04, p<0.01; *mPer3*:t = 6.03, p<0.0001) (Figure 2A). Interestingly, *mPer1* and *mPer3* were also upregulated by chronic cocaine in the NAc *(mPer1*: t = 2.24; p<0.05; *mPer3*:t = 2.1, p<0.05) while *mPer2* was not regulated in this region at this timepoint (Figure 2B). We saw no changes in the expression of the *mCry* genes by these treatments in either brain region (data not shown), suggesting that the paradigms employed selectively affect the *Per* genes.

These regional changes in mRNA expression following chronic cocaine exposure are also seen at the protein level as assessed by western blot analysis in a separate group of animals, indicating that they likely result in altered PER function (NAc: mPER: t = 5.33, p<0.001; mPER3: t = 8.627, p<0.0001: CP: mPER1: t = 3.14, p<0.01; mPER2: t = 3.83, P<0.01; mPER3 t = 6.277, p<0.0001), Figure 2C and 2D). Again, we saw no difference in mPER2 protein levels in the NAc at this timepoint. This regional difference in expression is interesting since the NAc is thought to be involved in drug reward and overall hedonic state while the CP is more involved in the habitual and compulsive behaviors associated with addiction and other psychiatric disorders [33]. These results also suggest that the regulation of these genes may occur through different second messenger pathways and may modulate different behavioral responses associated with mood and addiction.

### Cocaine treatment is associated with an increase in NPAS2 binding, but not CLOCK binding to the *Period* gene promoters in the CP and NAc

**Figure 3:**
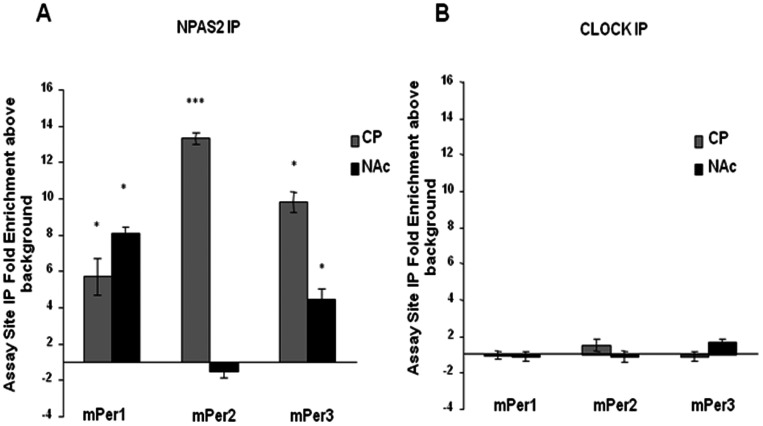
Binding of NPAS2 and CLOCK to the *Per* promoters in striatal regions. ChIP assays were performed with antibodies specific to CLOCK, NPAS2, and acetylated histone H3. IgG was used as a negative control. This was followed by real-time PCR analysis using primers specific to the *mPer1*, *mPer2*, or *mPer3* promoters.(A) ChIP assays were performed in NAc and CP tissue using an antibody for NPAS2 in animals treated chronically with cocaine (15 mg/kg, 7 days) or saline. Shown are the fold changes in cocaine treated animals versus saline treated animals. (B) ChIP assays were performed in NAc and CP tissue using an antibody for CLOCK in animals treated chronically with cocaine (15 mg/kg, 7 days) or saline. Shown are the fold changes in cocaine treated animals versus saline treated animals (Fold ±1). **p<0.01, ***p<0.001 by t-test (cocaine vs. saline), n = 6.

To better understand the molecular actions of repeated cocaine administration on the regulation of *Per* gene expression, we employed chromatin immunoprecipitation assays (ChIP) to determine if NPAS2 and CLOCK are binding to the promoter regions of the *Per* genes in striatal regions, and if cocaine treatment affects this binding. C57BL/6J mice were administered either chronic cocaine (15 mg/kg, i.p., once/day, 7 days) or saline. ChIP assays using CP and NAc tissue were then performed with antibodies directed against either NPAS2 or CLOCK. Chronic cocaine treatment produced a dramatic and selective increase in the binding of NPAS2, but not CLOCK, to all three *Per* gene promoters in the CP (*mPer1* promoter*:* t = 4.72, p<0.001; *mPer2* promoter: t = 32.79, p<0.001; *mPer3* promoter: t = 13.78, p<0.001; Figure 3A and 3B). There was also a significant increase in binding of NPAS2 to the *mPer1* and *mPer3* promoters in the NAc with no increase in CLOCK binding (*mPer1* promoter: t = 17.31, p<0.001; *mPer3* promoter: t = 4.79, p<0.01; Figure 3A and 3B). Interestingly, there was no increase in the binding of NPAS2 to the *mPer2* promoter in the NAc following cocaine treatment, which correlates with the lack of induction of this gene or protein by this treatment. These results suggest that chronic cocaine treatment leads to a selective induction of NPAS2 binding at certain *Per* gene promoters leading to an increase in their expression.

### A mutation in NPAS2 affects the induction of the *Period* genes after cocaine

**Figure 4:**
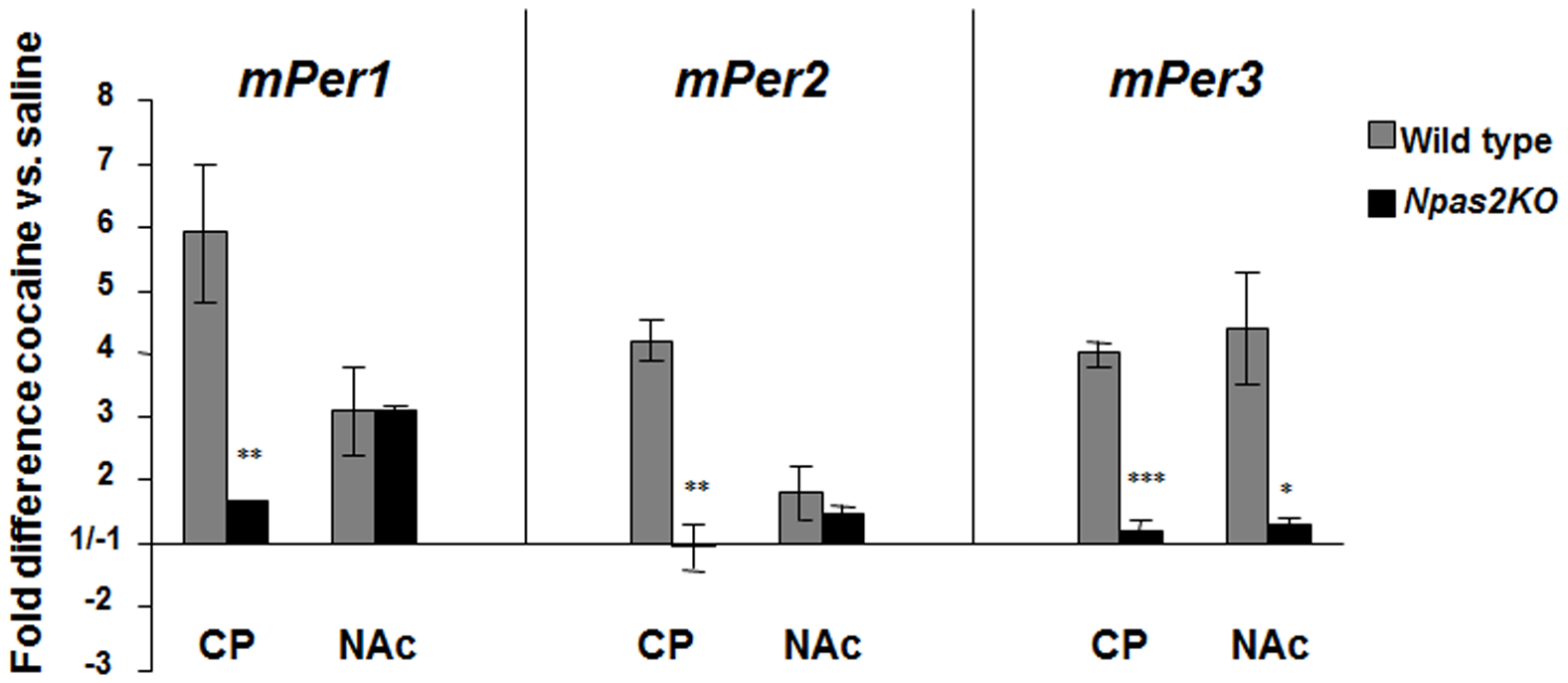
Effect of the *Npas2* mutation on *Per* gene induction following cocaine. Real-time PCR analysis of *mPer1, mPer2, and mPer3* expression in the CP and NAc following chronic treatment (15 mg/kg, 7 days) with saline or cocaine in wild type and *Npas2* mutant mice. Shown are the fold changes in cocaine treated animals relative to saline treated animals. *p<0.05, **p<0.01, ***p< 0.001 by t-test. n = 5.

To determine if NPAS2 is involved in the induction of these genes following chronic cocaine administration, we gave chronic cocaine (15 mg/kg, i.p. 7 days) or saline to the *Npas2* mutant mice and measured levels of *Per* gene expression 24 hours after the last treatment. Induction of the *Per* genes in the CP after chronic cocaine fail to occur if *Npas2* is disrupted (*mPer1:* t = 5.41, p<0.001; *mPer2:* t = 7.65, p<0.001; *mPer3:* t = 14.42, p<0.001; Figure 4). However, while the induction of *mPer3* is also diminished in the NAc, the induction of *mPer1* in this region is unchanged (*mPer3:* t = 3.09, p<0.05; Figure 4). *mPer2* levels in the NAc were unaffected since they are not induced by cocaine in wild type mice. This suggests that NPAS2 is necessary for the induction of all three *Per* genes in the CP following cocaine treatment and *mPer3* in the NAc, however, it is not necessary for the induction of *mPer1* in the NAc. We did not see a significant reduction in baseline *Per* gene expression levels in the *Npas2* mutant mice versus wild type controls at this timepoint. The expression levels of these genes in striatum are normally low at the time of day that these experiments were performed (between ZT 3–6) [3], [19], [30], therefore a further reduction in expression is difficult to assess, and has not been seen in previous studies [19]. However, the peak levels of *mPer2* expression (occurring during the subjective night) are dampened in these mice under baseline conditions, selectively in regions of the brain that express NPAS2 [19].

### Chronic cocaine treatment alters molecular rhythms in striatal regions

**Figure 5:**
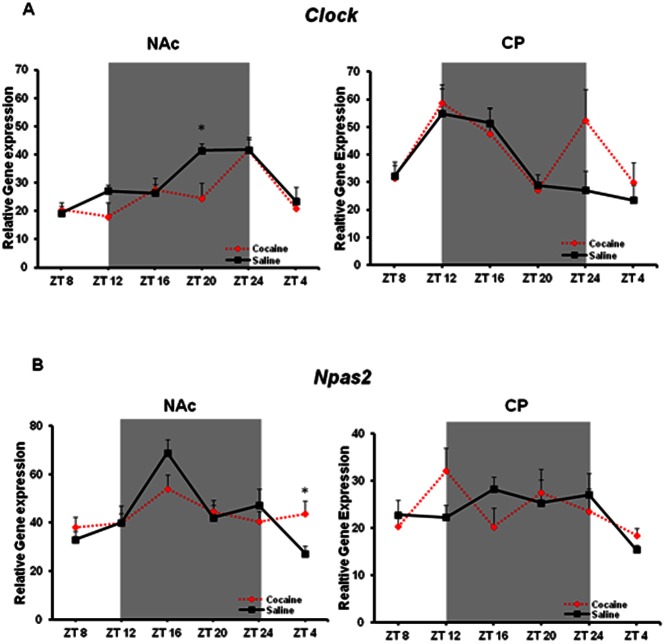
Chronic cocaine alters *Npas2* rhythmic expression only in the NAc, whereas *Clock* is unchanged. (A) *Clock* gene expression (mean ± SEM, n =  6–9) in the NAc and CP is unaltered by chronic cocaine (B) *Npas2* rhythmicity in the NAc is abolished following chronic cocaine and a significant upregulation was observed at ZT 4 (*p<0.05). Dark background indicates lights-off.

Since all of the gene expression studies were performed at a single timepoint, it was of interest to know whether chronic daytime cocaine treatment leads to an increase in *Npas2* and *Per* gene expression across the light/dark cycle while maintaining a normal rhythm, or if it is part of an alteration in their molecular rhythms. Mice were treated with cocaine (15 mg/kg) or saline i.p. for 7 days at ZT 6 (1pm). Animals were sacrificed starting 26 hrs after the last injection (ZT8, 3pm) and subsequently every 4 hrs until ZT4 (11am). The last timepoint is exactly 2 hrs prior to drug administration time and thus, might provide some insight into possible entraining effects of the drug. *Clock* exhibits diurnal rhythmicity in both striatal regions under saline conditions (F_(5,41)_ = 5.389, p = 0.0007 NAc; F_(5,36)_ = 4.216, p = 0.0042 CP), and rhythmicity is maintained following chronic cocaine treatment (F_(5,40)_ = 6.120, p = 0.0003 NAc; F_(5,35)_ = 2.937, p = 0.0257 CP) (Figure 5A). A two-way ANOVA revealed a lack of effect of treatment and interaction, but a highly significant effect of time in both regions (F_(5,81)_ = 9.82, p<0.0001 NAc; F_(5,69)_ = 5.83, p = 0.0002 CP). Even though no interaction effect was observed, a significant difference was found at ZT 20 in the NAc (*p<0.05) and a nonsignificant trend toward up-regulation was observed at ZT 24 in the CP.

In contrast, the robust diurnal rhythmicity of *Npas2* in the NAc under saline conditions is abolished following chronic cocaine treatment (F_(5,40)_ = 6.720, p = 0.0001 saline; F_(5,43)_ = 1.120, p = 0.3641 cocaine). There was no significant rhythm in*Npas2* expression in the CP under both treatment conditions. A significant upregulation was observed at ZT 4 in the NAc following cocaine treatment, but not the CP (*p<0.05; Figure 5B). Interestingly, no difference was observed at ZT8, just a few hours after a timepoint similar to that in Figure 1, suggesting that this effect dissipates by ∼26hrs post-injection, but returns a few hours prior to drug administration.

**Figure 6:**
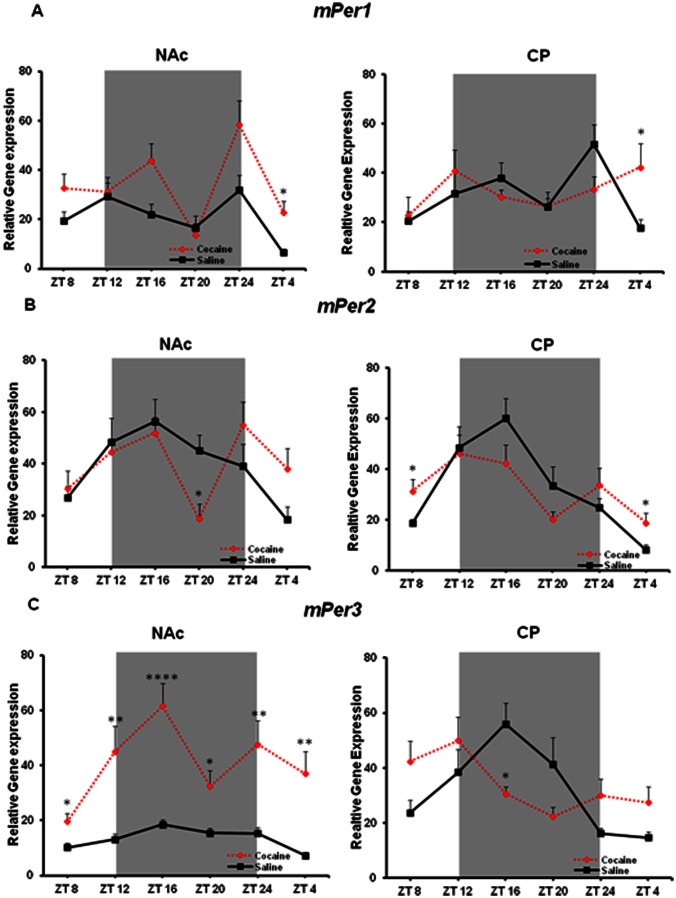
Chronic cocaine alters diurnal expression of *Per* genes in the NAc and CP. (A) Cocaine alters *mPer1* expression (mean ± SEM, n = 6–9) in the NAc and CP. Rhythmicity of *mPer1* is maintained in the NAc but abolished in the CP following cocaine treatment. A significant upregulation at ZT4 was observed in both regions (*p<0.05). (B) *mPer2* rhythmicity is unaltered in the NAc but disrupted in the CP following cocaine. A significant upregulation at ZT 4 was observed only in the CP (*p<0.05). (C) While rhythmicity is maintained, *mPer3* is highly upregulated in the NAc. Cocaine disrupts rhythmicity of *mPer3* in the CP.

*mPer1* exhibits diurnal rhythmicity in both striatal regions under control conditions (F_(5,27)_ = 3.575, p = 0.0131 NAc; F_(5,36)_ = 3.826, p = 0.007 CP; Figure 6A). Following chronic cocaine, this rhythmicity is lost in the CP, but maintained in the NAc (F_(5,33)_ = 4.862, p = 0.0019). A two-way ANOVA revealed a highly significant effect of treatment (F_(1,60)_ = 12.18, p = 0.0009) (F_(5,60)_ = 6.52, p<0.0001) in the NAc. There was also a significant effect of treatment (F_(5,67)_ = 2.48, p = 0.0401) in the CP. Again, an upregulation of *mPer1* was observed following cocaine in both regions at ZT 4 (*p<0.05).

*mPer2* is rhythmic in the NAc and this rhythm is mostly preserved following cocaine except for a significant downregulation at ZT 20 (F_(5,34)_ = 3.839, p = 0.0073 saline; F_(5,35)_ = 3.589, p = 0.01 cocaine; Figure 6B). However, in the CP, the amplitude of *mPer2* rhythms are significantly dampened following cocaine treatment (F_(5,35)_ = 8.273, p<0.0001 saline; F_(5,38)_ = 2.005, p = 0.1 cocaine). In the NAc, a two-way ANOVA confirmed a significant drug interaction effect (F_(5,69)_ = 2.47, p = 0.00404) and a highly significant time effect (F_(5,69)_ = 5.08, p = 0.0005). There was also a highly significant effect of time (F_(5,72)_ = 9.13, p<0.0001) in the CP. An upregulation at ZT4, similar to that previously observed, and ZT8 were observed in the CP and not the NAc following cocaine (*p<0.05).

*mPer3* displays diurnal rhythmicity in both regions under saline conditions (though the amplitude is low in the NAc) (F_(5,40)_ = 5.176, p = 0.0009 NAc; F_(5,33)_ = 5.077, p = 0.0015 CP; Figure 6C). After cocaine treatment, expression of *mPer3* is increased and rhythmicity is maintained in the NAc (F_(5,36)_ = 4.135, p = 0.0045) however the rhythm is significantly dampened in the CP (F_(5,35)_ = 2.343, p = 0.0617). A two-way ANOVA revealed in the NAc a significant interaction effect (F_(5,76)_ = 3.16, p = 0.0121), a highly significant drug treatment effect (F_(1,76)_ = 93.59, p<0.0001), and a highly significant effect of time (F_(5,76)_ = 6.11, p<0.0001). No such effects were detected in the CP; however, there seemed to be a trend towards a shift in the expression of *mPer3*, peaking during the day and not the dark phase. All timepoints in the NAc showed a significant upregulation by cocaine when compared to saline (*p<0.05, **p<0.01, ****p<0.0001). In the CP, a significant timepoint difference was observed only at ZT 20 (*p<0.05).

## Discussion

This study found that specific circadian genes/proteins are altered by cocaine treatment in striatal regions. Some of these alterations are not just timepoint-specific effects but rather reflect a change in the overall rhythmic expression of these genes. Both NPAS2 and CLOCK proteins are expressed in the NAc and CP, however, only *Npas2* expression is induced by cocaine in these regions. Furthermore, both NPAS2 and CLOCK bind to the *Per* gene promoters in striatal regions, though chronic cocaine treatment only affects the binding of NPAS2 at these promoters. Interestingly, the *Per* genes were differentially regulated by acute and chronic cocaine in both NAc and CP. Similar to previous studies, *mPer1* is induced by acute cocaine while *mPer2* is induced by chronic cocaine [6], [7]. Thus, it appears that *mPer1* is differentially regulated in response to cocaine and may be involved in some of the acute effects of the drug while *mPer2* and *mPer3* may be more involved in the chronic effects of the drug. Interestingly,*mPer1* displays the kinetics of an immediate-early gene in response to light in the SCN while *mPer2* responds more slowly [34]. The function of *mPer3* in the SCN appears to be minimal and outside of the central functions of the molecular clock [35]. Therefore, the induction of *mPer3* by cocaine may have completely different consequences than the induction of the other *Per* genes. Interestingly, Pieper *et al* identified about twice as many genetic variations in *hPer3* than *hPer1* in human subjects with psychiatric disorders, suggesting that *Per3* may be more involved in mental illness than *Per1* [36].

The observed cocaine-induced regulation of all three *Per* genes in the CP, and *mPer2* and *mPer3* in the NAc, was found to be mediated through NPAS2, since the response was abolished in *Npas2* mutant mice. Another interesting finding is that the increase in *mPer1* expression by cocaine in the NAc is not prevented in the *Npas2* mutant mice even though cocaine leads to an increase in NPAS2 binding at the *mPer1* promoter in this region. *mPer1* and *mPer2* both have CREB binding sites in their promoters, however only *mPer1* seems to be responsive to CREB regulation [37]. Therefore, it is possible that the increase in *mPer1* expression in the NAc with cocaine treatment is due to both NPAS2 and CREB, and that CREB is sufficient to induce this activity in the absence of NPAS2. This is also supported by the fact that a dopamine D1, but not D2 type, receptor antagonist can block the induction of *mPer1* in the CP by acute methamphetamine treatment and a D1, but not D2, receptor agonist can induce *mPer1* expression in striatal neurons [7], [38]. It is interesting that we find *mPer2* to be altered in the CP and not the NAc with chronic cocaine treatment. The NAc is associated with the regulation of drug reward, the processing of emotional stimuli, and sensitized locomotor responding to psychostimulants, while the CP has been linked to the regulation of motor activity, as well as the control of habitual and compulsive behaviors [5], [33], [39]. Thus PER2 may be more involved in behaviors controlled by the CP. Indeed previous studies have found that a line of mice lacking *mPer2^ Brdm1^* show no differences in the conditioned place preference for cocaine, but have an increase in alcohol seeking behavior and self-administration, which may involve the CP [12], [15]. However, it is possible that PER2 may have a role in counteracting NAc mediated- behaviors like sensitized locomotor responding, since *mPer2^ Brdm1^*mice hypersensitize to cocaine [5].

Rhythmic expression and function of circadian genes or components of the clock are also under the influence of posttranscriptional and posttranslational modifications [40], which could explain the apparent discrepancy between changes in diurnal expression when comparing *Npas2* and the *Per* genes in the CP. Indeed, NPAS2 binding is increased at the *Per* promoters following chronic cocaine, suggesting a possible posttranslational modification of NPAS2 that could affect *Per* expression. Alterations in clock gene expression in the striatum in response to drug treatment has profound behavioral effects on the animal in terms of altering the sleep/wake and activity cycle, as animals increase their locomotor activity in anticipation of the drug [3], [41]. This anticipatory behavior is observed at least 2 hrs prior to the time of drug administration. Furthermore, changes in circadian gene expression in reward related regions like the NAc may reinforce the importance of cocaine over other stimuli at certain times of day, and lead to an increased craving at the time when cocaine is expected. Thus, it was of interest to investigate the effect of chronic cocaine on the rhythmic expression of these genes in striatal regions. The purpose for this was two-fold. First, assess if the regulation already observed by cocaine was a timepoint-specific effect or if it was an overall change in the gene’s rhythmic expression; and secondly, determine if there was an anticipatory effect in the expression of these genes that could presumably underlie behavioral anticipation. Interestingly, cocaine treatment disrupted rhythmic expression of *Npas2* and led to an overall increase in *mPer1* and *mPer3* in the NAc. In the CP, chronic cocaine led to a disruption in the rhythmic expression of all the *Per* genes. *Clock* was relatively unaffected, supporting the idea that in striatal regions, NPAS2, and not CLOCK, is a key player in mediating cocaine responses. A pilot study was performed to determine if there was any effect of cocaine in rhythmic expression of *mPer2* in the SCN. No such regulation was observed, save for a slight reduction in the peak of the rhythm (data not shown). The up-regulation of certain genes at ZT 4 (2 hrs prior to administration time) suggests a possible rise in circadian gene expression that could drive anticipatory locomotor events. It is important to note that this timepoint is 46 hours after the animal’s last cocaine injection, making this a persistent effect just like the one observed behaviorally, which persists for a couple of days after drug has been withheld. It is, however, unclear whether this increase in gene expression leads to the increased locomotor activity or vice-versa. Still, it is possible that effects observed are also due to acute cocaine withdrawal, as previous studies have found that by 36 hours of withdrawal rhythmic expression of circadian genes in striatal regions are altered [42]. For behavioral entrainment to occur, a manipulation or regulation of the circadian machinery must occur by photic and/or non-photic cues. Thus, it is likely that this alteration in core circadian genes in the striatum (NAc and CP) would in turn alter dopaminergic neurotransmission, which is known to be involved in locomotion and arousal. Therefore it is possible that these changes in core circadian gene expression underlie the increase in anticipatory locomotor activity observed in behavioral entrainment to drugs of abuse. It would be interesting to measure how long this effect lasts, since studies have shown that twice daily morphine injections for 7 days cause significant alterations in circadian gene rhythmic expression, as well as circadian patterns of hormones and peptides, that persist for up to 60 days after withdrawal [41]. Further studies will look into the effects in gene expression between different times of treatment and times of data collection. Additionally, future studies will determine the impact of altered circadian gene rhythmicity on neuronal activity and plasticity within individual striatal regions.
